# A Homotopic Direct Collocation Approach for Operational-Compliant Trajectory Design

**DOI:** 10.1007/s40295-022-00351-x

**Published:** 2022-11-09

**Authors:** Alessandra Mannocchi, Carmine Giordano, Francesco Topputo

**Affiliations:** Department of Aerospace Science and Technology, Politecnico di Milano, Milan, Italy

**Keywords:** Homotopy, Continuation, Direct transcription and collocation, M-ARGO CubeSat

## Abstract

Stand-alone deep-space CubeSats are the future of the space sector. For limited budget reasons, these spacecraft need to follow operational-compliant (OC) trajectories: transfers with thrusting and coasting periods imposed at pre-defined time instants. Traditional trajectory optimisation algorithms exhibit convergence problems when handling discontinuous constraints. In this work, a homotopic direct collocation approach is presented. It employs a continuation algorithm that maps the classical bang-bang trajectory of a fuel-optimal low-thrust problem into an OC solution. M-ARGO CubeSat mission is considered as case study for validation, including a realistic thruster model with variable specific impulse and maximum thrust. The trajectories computed with the developed algorithm are compared with non-operational-compliant solutions. Our algorithm produces transfers similar to the optimal solutions with no operational constraint, both in terms of thrusting profile and propellant mass.

## Introduction

The deep-space sector is becoming progressively accessible: while traditional missions were designed with large budgets, in the last years we are witnessing a significant reduction of costs. An expression of this trend is the rapid development of interplanetary CubeSat technology [32]. Several released-in-situ, deep-space CubeSat missions are expected to be launched in the next years by ESA (e.g., LUMIO [7], Milani [12], Juventas [16]). Another class of stand-alone interplanetary CubeSats will travel to their final destination without the need of a carrying mothership. The Miniaturised Asteroid Remote Geophysical Observer (M-ARGO) [31] will be the first European CubeSat to perform a similar mission.

The reduced size of miniaturized spacecraft imposes skeletal budgets on their systems [24] (e.g. in terms of power, propellant, and data handling). The high specific impulse makes electric propulsion a good candidate for these probes [27]. Still, electric propulsion requires a significant on-ground flight dynamics effort with regular navigation and guidance operations. To overcome theses issues, spacecraft, and especially CubeSats, will be required to follow operational-compliant (OC) trajectories, consisting of a repetition of a regular pattern of alternating thrusting and coasting arcs (*duty cycles*). Operations, as communication, ground-based orbit determination and correction, or scientific experiments, will be performed during coasting arcs. They will also ease ground operations, and thus in turn lower the cost of flight dynamics team.

Electric propulsion trajectories are determined through the formulation of a low-thrust optimal trajectory problem (LOTP), a specialisation of the optimal control problem (OCP) for time-continuous systems [6, 18]. No analytic solutions exist for this optimal problem, but several numerical techniques, traditionally divided in direct and indirect methods [4], have been developed to solve the LOTP. Indirect methods [20, 28] aim at finding the solution of the necessary optimality conditions derived by calculus of variations. Instead direct methods [10, 17] transcribe the optimal control problem into a parameter optimisation problem, and then use nonlinear programming (NLP) to find the optimal solution. Both of them are solved by means of gradient methods [25], which require the explicit use of the first and sometimes second order derivatives of the problem. For this reason, the functions of the LOTP are required to be differentiable, and thus, continuous [5].

Duty cycles, them being time-dependent and discontinuous constraints, are thus not straightforwardly introduced in both methods. They yield a small convergence radius, preventing the convergence of the solver [22]. Homotopy, or continuation, is a suitable method to deal with discontinuous structures. The homotopic approach allows solving the original, difficult and discontinuous problem, starting from an easier and affordable one [19, 34]. In particular, it has been applied to indirect methods to overcome discontinuity problems as bang-bang control in fuel optimal solutions [3, 11, 35].

In this paper, a new technique, here called* homotopic direct collocation* (HDC) algorithm, is derived to generate OC trajectories by enforcing duty cycles through a homotopic approach applied to a Hermite-Simpson direct collocation method, making the problem only gradually discontinuous. This is achieved imposing to a fuel optimal not OC trajectory heavier weights to the intervals corresponding to coasting arcs until an OC trajectory is obtained. In the end, HDC tries to answer to the question whether is possible to map a real-fuel-optimal but not-OC solution, into a fuel sub-optimal but OC solution, and, in case, how to achieve this mapping. To the authors’ knowledge, the HDC is the first attempt of applying an homotopic approach to a direct collocation algorithm. Usually, the presence of duty cycles is considered in early trajectory design phases with the approximation of imposing a minor value of the maximum thrust available on board. The benefits of our algorithm is that it is not an approximation, thus it provides directly the correct solution with both the thrust and the control angles profiles, and that it is a general approach, allowing the modeling of each kind of duty cycle that is needed to be imposed. The results obtained from HDC are applied to the case of M-ARGO mission, as solution to the needs of the trajectory design of this peculiar mission. The interplanetary transfers are computed using a realistic thruster model, which includes variable maximum thrust and specific impulse. HDC solutions are shown to have thrusting profiles and required propellant mass similar to the solutions without the duty cycle constraint.

The paper is organized as follows. In Sect. 2 a brief review on the low-thrust optimal trajectory problem is given. In Sect. 3 the structure and the main issues of the optimal solution of the problem are discussed. The presentation of the HDC algorithm is in Sect. 4. A first example for the validation of HDC is presented in Sect. 5. Results applied to M-ARGO mission and conclusions are presented in Sects. 6 and 7, respectively.

## The Low-Thrust Optimal Trajectory Problem

Consider the following dynamics for a spacecraft in spherical coordinates

1 $$\dot{{\textbf{x}}}(t) = {\textbf{f}}({\textbf{x}}, \quad {\textbf{u}}, \quad t) = \left[\begin{array}{c} v_r\\ \frac{v_{\theta }}{r \cos {\phi }} \\ \frac{v_{\phi }}{r} \\ {\textbf{a}}_{SRP} + {\textbf{P\;a}}_G + {\textbf{S}}[v_{r} \quad v_{\theta } \quad v_{\phi }]^T + {\textbf{a}}_T \\ -\frac{T}{I_{sp} g_0}\end{array}\right]$$

where **x** is the state vector

2 $${\textbf{x}}(t) = [{\varvec{r}}, \quad {\varvec{v}}, \quad m] = [r, \quad \theta , \quad \phi , \quad v_r, \quad v_{\theta }, \quad v_{\phi }, \quad m]$$

where $\phi$ and $\theta$ are the azimuth and elevation angles in J2000 reference system respectively, and *m* is the mass of the spacecraft. The control vector **u**(*t*) is a function of time for low-thrust propulsion models, and is expressed as

3 $${\textbf{u}}(t) = [T, \quad \alpha , \quad \beta ]$$

where *T* is the thrust magnitude, $\alpha$ is the in-plane angle such that $\alpha \in [-\,180, \, 180]$ deg, and $\beta$ is the out-of-plane thrust angle such that $\beta \in [-\,90, \, 90]$ deg. The thrust magnitude is equal to the product between the maximum thrust $T_{max}$ and the throttle factor *u*, such that $u = 0$ means null thrust, and $u = 1$ means maximum thrust. The gravitational pulling ${\textbf{a}}_G$ in Eq. (1) describes a full-ephemeris model according to which

4 $${\textbf{a}}_G = {\varvec{g}}({\varvec{r}}) + {\textbf{a}}_{G, i} = -\frac{\mu _{\odot }}{r^3}{\textbf{r}} - \mathop{\sum}\limits_{i \in P}\mu _i \left( \frac{{\textbf{r}}_i}{r_i^3} - \frac{{\textbf{r}}_i - {\textbf{r}}}{\Vert{\textbf{r}}_i - {\textbf{r}}\Vert^3} \right)$$

where **r** is the position vector of the spacecraft, $\mu _{\odot }$ is the gravitational constant of the Sun, and thus the first term of Eq. (4) is the primary acceleration due to the Sun. The other terms in the Eq. (4) model the gravitational accelerations given by the 8 planets of the Solar System, represented by the set *P*: $\mu _{i}$ is the planetary gravitational constant of the *i*-th planet and ${\textbf{r}}_i$ is the position vector of the spacecraft with respect to it. The numerical formulation of Eq. (4) employs the method by Betts (see Appendix F of [9]) to avoid numerical errors in computing the difference between nearly equal numbers. The SRP acceleration ${\textbf{a}}_{SRP}$ is expressed as

5 $${\textbf{a}}_{SRP} = \frac{Q\; A}{m}\frac{{\textbf{r}}}{r^3}$$

where *Q* is the solar radiation pressure constant and *A* is the Sun-projected area. The thrusting acceleration ${\textbf{a}}_T$ is represented by

6 $${\textbf{a}}_T = \frac{T}{m}\varvec{\alpha } = \frac{T_{max} u}{m} \left[\begin{array}{c} \sin {\alpha } \cos {\beta } \\ \cos {\alpha } \cos {\beta } \\ \sin {\beta } \end{array}\right]$$

Eventually, in Eq. (1) **P** is a rotation matrix and **S** is a skew-symmetric matrix mapping the velocity vector, defined as

7 $${\textbf{P}} = \left[\begin{array}{ccc} \cos {\phi } \cos {\theta } & \quad \cos {\phi } \sin {\theta } &\quad \sin {\phi }\\ -\sin {\theta } &\quad \cos {\theta } &\quad 0 \\ -\sin {\phi } \cos {\theta } & -\sin {\phi } \sin {\theta } &\quad \cos {\phi } \end{array}\right] \quad {\textbf{S}} = \left[\begin{array}{ccc} 0 &\quad {\dot{\theta }}\cos {\phi } &\quad {\dot{\phi }}\\ -{\dot{\theta }}\cos {\phi } &\quad 0 &\quad {\dot{\theta }}\sin {\phi } \\ -{\dot{\phi }} &\quad -{\dot{\theta }}\sin {\phi } &\quad 0 \end{array}\right]$$

where ${\textbf{P}}$ is used to convert ${\textbf{a}}_G$ in spherical coordinates while ${\textbf{S}}$ is used to write the dynamics in a more compact way. The LOTP aims at computing the optimal control function ${\textbf{u}}(t)$ that minimizes a scalar cost function *J*. In space trajectories design, *J* is commonly represented by the time of flight, ToF, or the propellant mass, $m_p$, required. When the objective is to save the propellant mass $m_p$, *J* is referred as $J_f$, and the LOTP is called fuel-optimal problem (FOP):

8 $$J_f = \int \limits _{t_i}^{t_f} \frac{T(t)}{I_{sp}(t)} \, \text{d}t$$

As discussed in Sect. 1, no analytic solutions exist for the LOTP, but there exist several numerical techniques to solve it. Direct methods firstly discretised the problem imposing the dynamical constraints, and then find the optimal trajectory through the solution of a NLP problem. In particular, direct collocation methods transcribe the LOTP into a parameter optimisation problem enforcing the dynamics with numerical integration schemes, such as Euler or Hermite-Simpson [29], allowing the transcription of differential constraints into algebraic ones. In this work it has been employed as starting point the software *DIRETTO* [30], a low-thrust trajectory design tool developed at Politecnico di Milano, which solves the LOTP with a Hermite-Simpson direct collocation method and exploits Ipopt^1^ [33] as NLP solver.

## Structure of the Optimal Solution

The necessary conditions for optimality of the solution of the FOP are derived by introducing the Lagrange multipliers, or costates, $\varvec{\lambda } = [\varvec{\lambda }_r, \, \varvec{\lambda }_v, \, \lambda _m]$ associated to the state ${\varvec{x}} = [{\varvec{r,v}}, \quad m]$. Indicating the optimal thrust direction as $\varvec{\alpha }^{\ast}$, it can be shown [8, 23] that it is

9 $$\varvec{\alpha }^{\ast} = -\frac{\varvec{\lambda }_v}{\lambda _v} \quad \text{if} \quad \lambda _v \ne 0$$

**Fig. 1:**
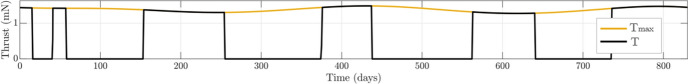
The typical bang-bang fuel-optimal thrusting profile

Inserting this into the necessary conditions for optimality of the LOTP, the Euler–Lagrange equations [6, 26] it can be demonstrated that the optimal throttle factor, $u^{\ast}$, is determined as

10 $$u^{\ast} = \left\{ \begin{array}{ll} 0 & \quad \text{if} \; S >0 \\ 1 &\quad \text{if} \; S <0 \end{array}\right.$$

where *S* is the switching function, $S = -\lambda _v \frac{I_{sp}g_0}{m} - \lambda _m + 1$. Accordingly to this Pontryagin’s maximum principle (PMP) [21], the fuel-optimal control has a bang-bang profile, having a piece-wise discontinuous structure, either zero or maximum value, as in Fig. 1. In particular this represents the fuel optimal thrust profile for the transfer of M-ARGO CubeSat to asteroid 2011 MD starting the June 5, 2023, and lasting 830 days (see Sect. 6 for more details on the statement of the problem).

The solution of a FOP as in Fig. 1 is fuel-optimal, but not OC, showing long thrusting and coasting arcs. A deep-space spacecraft can not thrust for long time periods, because it could not perform any other task in the meanwhile, as communicating, controlling its trajectory, and so on. Even for classic spacecraft, thrusting for long time periods means accumulating remarkable errors along the trajectory, and thus ideally, some coasting phases have to be inserted in between thrusted-arcs.

## The Homotopic Direct Collocation Algorithm

A duty cycle, with the duration of *n* days, binds the control in an alternation of thrusting and coasting arcs, with the duration of $n - m$ and *m* days respectively, as follows

11 $$u = \left\{ \begin{array}{ll} [0, \, 1] &\quad \hbox{if} \; t \in [0, \, t_{n - m}] \;\text{days} \\ 0 &\quad \hbox{if} \;t \in [t_{n - m}, \, t_m] \; \text{days} \end{array}\right.$$

where *n* and *m* are the design parameters for the duty cycle. Looking at the control profile in Fig. 1, it can be noted that it *already* presents an alternation of thrusting and coasting regimes. Since optimal solutions like this are produced by the solver in order to minimize $J_f$. If $J_f$ is written such that the control of the *m*th day of each duty cycle is less convenient in terms of propellant with respect to the controls of the remaining $(n - m)$ days, the optimiser can be driven to exclude it from the optimal thrusting profile. The idea is to overweight in $J_f$ the time intervals in which the thrust is not desired by considering penalty factors, or weights. To achieve this, we firstly discretised the integral of $J_f$ in Eq. (8) over each time interval of duration $h_k$, and then modified it with penalty weights $w_k$. Since for electric thrusters the specific impulse $I_{sp}$ varies with the input power, and so with the distance from Sun, this variation should be considered in the cost function. The controls are linearly interpolated in each segment, such that $T_k = \frac{1}{2}(T(t_k) + T(t_{k + 1}))$ and $I_{sp,k} = \frac{1}{2}(I_{sp}(t_k) + I_{sp}(t_{k + 1}))$.

12 $$J_f= \sum _{k=1}^{N_s} \frac{T_k h_k}{I_{sp,k}}$$

13 $$J_f= \sum _{k=1}^{N_s} \frac{T_k h_k w_k}{I_{sp,k}}$$

In Eqs. (12, 13) $N_s$ is the number of interval $[t_k, \, t_{k + 1}]$ considered for the Hermite-Simpson collocation. For each of them, weights $w_k$ are unitary during thrusting arcs, while they are selected higher during coasting arcs.

**Fig. 2:**
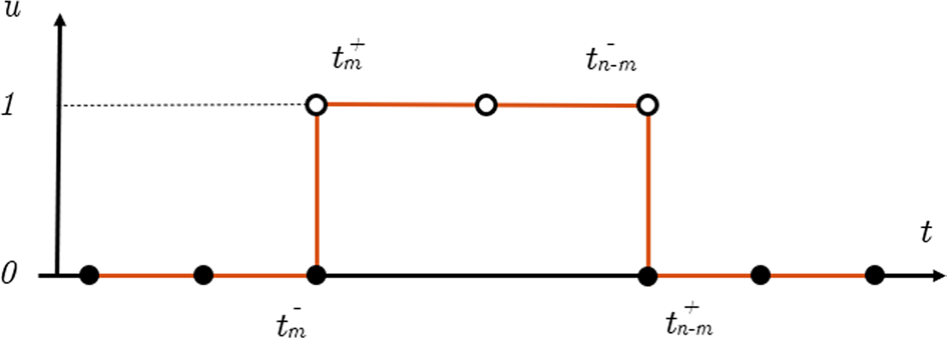
Instantaneous switching of the bang-bang control

The discontinuity posed by the instantaneous switching of the engine from on to off and viceversa in a bang-bang control can be difficult to be solved. Consider Fig. 2.

**Fig. 3:**
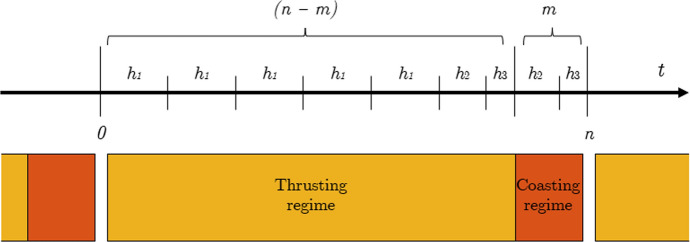
Duty cycle with the not-equally distributed time grid

Theoretically, on each node $t_m$, corresponding to the end of the non-thrusting regime of a duty cycle, two different values of the control should be considered, one right before that instant, at $t_m^-$, with $u = 0$, and one right after it, at $t_m^+$, with $u > 0$. The same problem, with inverted values of the control, is present at $t_{(n - m)}$, corresponding to the end of the thrusting regime. Two different values of the control can not be enforced on a single node, but it is allowed to introduce two nodes very close each other. Thus a not-equally distributed time grid has been considered, as in Fig. 3.

Given the nature of the grid, it is possible to properly model the thrusting and coasting phases. For each duty cycle, considering the vectors of the time intervals and of the corresponding weights

14 $${\textbf{h}} = \left[\underbrace{h_1, \quad h_1, \quad h_1,\quad \dots \quad h_1,}_{\text {(n-m-1)}} \quad h_2, \quad h_3, \quad h_2, \quad h_3\right]$$

15 $${\textbf{w}} = \left[\underbrace{1, \quad 1, \quad 1, \quad \dots \quad 1,}_{\text {(n-m)}} \quad w_a, \quad w_b, \quad w_c\right]$$

the weights have been selected such that $1< w_a< w_c < w_b$. Indeed, since the first interval $h_3$ belongs to the last day of thrusting of a duty cycle, it should not be penalised with a weight $w_a$ high as the ones in the coasting day. Similarly, the last interval $h_3$ belongs to a coasting day, but since it is linked by the linear interpolation to the control of the first day of the following duty cycle, its weights $w_c$ should model the switching-on of the thruster, while not being so penalised as the control weighed by $w_b$. Moreover, this selection of the weights ease the convergence, smoothing the discontinuity of the control structure.

**Fig. 4:**
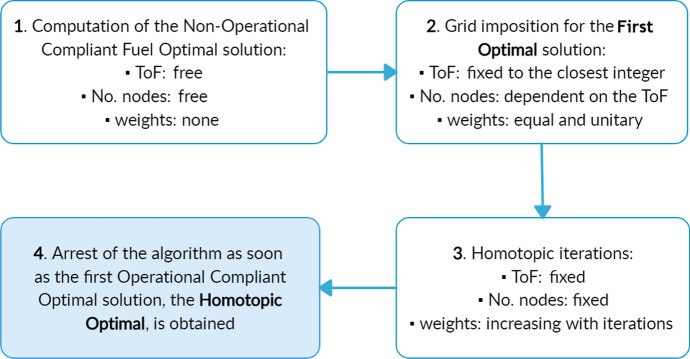
Scheme of the homotopic direct collocation algorithm

Due to numerical noise problem, the weights $w_k$ can not be imposed arbitrarily high in order to obtain an OC solution with a single iteration from a non-OC one. This would made the problem almost discontinuous from the very first iteration, so making it difficult to solve. For this reason, starting from a fuel-optimal not-OC solution as initial guess, the weights are introduced gradually increased at each iteration, up to when an OC solution is obtained. The HDC algorithm explicitly operates as shown in Fig. 4.

It can be summarised as:

1. firstly, a fuel non-OC optimal trajectory is computed using the Eq. (12), with an indicative number of nodes, an equally divided grid and a ToF left free to vary, in order to get a good first guess;
2. the above solution is used as the initial guess of a new optimisation using the Eq. (13), with all weights $w_k$ unitary, in order to impose the non-uniform time grid, and with the ToF fixed to the closest integer to the one computed at step 1. The definitive number of nodes of the grid is selected at this stage accordingly to the value of the ToF in order to have a correct time discretisation. The result is called First Optimal (FO) solution, and it requires the same $m_p$ of the first trajectory computed at step 1;
3. the solution of step 2 is selected to be the initial guess of a new optimisation using Eq. (13), where the weights $w_k$ are slightly increased in the time intervals during which the coasting phases are required to be imposed;
4. step 3 is repeated, each time considering the previously computed solution as initial guess and a slightly increased value of the weights in correspondence of intervals representing the coasting phases, until an OC solution is obtained: the final solution is called Homotopic Optimal (HO).

It has been experienced that if too high weights are introduced too early, the optimiser starts having convergence problems with the discontinuity of duty cycles. In case the convergence is not reached, the initial guess is re-fed to the optimiser, employing a slightly smaller value of the weights as compared to the failed value.

## A First Example

Considering Fig. 1, the long thrusting blocks for the solution of a FOP are yet located in the optimal location given a departure date and a ToF. The HDC idea is that the most convenient duty cycles of a solution both fuel-optimal and OC should be located around them, and not casually located along all the duration of the cruise. Thus, an optimal OC trajectory should be in aspect very similar to the not-OC solution, but with the long thrusting blocks interrupted when the coasting phases are imposed. The authority lost during them is expected to be recovered by HDC with thrusting arcs located immediately before and/or after the long thrusting blocks.

To test this intuition, as well as the use of the not-equally distributed grid, a first simple example has been assessed. Considering again the interplanetary trajectory of M-ARGO CubeSat to asteroid 2011 MD in Fig. 1, we want to force the solver to switch off the engine for 20 days around day 200. In order to achieve this, some weights $w_{l}$, for the day 1 and for the day 20 of the coasting arc, and $w_{h}$, for the remaining 18 days, have been selected such that $w_{l} < w_{h}$ after the first step. Starting from the FO solution in Fig. 1, 2 homotopic iterations summarised in Table 1 have been computed to create the 20-days-long coasting arc in the thrust profile.

**Table 1:** Iterations for the enforced coasting arc of 20 days

Iteration	${w_{l}}$	${w_{h}}$	${m_{p}}$ (kg)	${{\Delta }} {{m_{p}}} (\%)$
1-FO	1	1	1.26867	–
2	1.15	1.35	1.28696	1.44

**Fig. 5:**
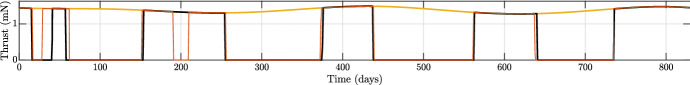
HDC recover the lost thrust lost changing the control profile

As it can be noted, the increase in required propellant mass is limited to 1.44%. In Fig. 5, the new thrusting profile, in red, has been reported in comparison with the initial FO solution, in black. The coasting arc around the day 200 of transfer is correctly imposed. The solver, to recover the lost thrust, changes the solution globally, elongating the duration of the second thrusting block, and slightly changing the profile around day 400 and day 600.

## Application to M-ARGO Mission

M-ARGO will be the first stand-alone CubeSat mission designed by the ESA aimed at targeting a near-Earth asteroid. It has the necessity of exploiting OC trajectories, and thus it has been used as study case to test the algorithm. All the assumptions about the thruster and the mission analysis are taken from [31]. The CubeSat will have a maximum wet mass of 28.2 kg, with the propellant mass constrained to be lower than 3.4 kg. The Sun-projected area and the reflectivity parameter have been assumed to be 0.3 m^2^ and 1.3, respectively.

To model the electric thruster it has been assumed that both the maximum thrust, $T_{max}$, and the specific impulse, $I_{sp}$, depend on the engine input power, $P_{in}$, which in turn depends on the distance of the spacecraft from the Sun, *r*. This dependence has been modeled with fourth-order polynomials:

16 $$T_{max}= a_0 + a_1P_{in} + a_2P_{in}^2 + a_3P_{in}^3 + a_4P_{in}^4$$

17 $$I_{sp}=b_0 + b_1P_{in} + b_2P_{in}^2 + b_3P_{in}^3 + b_4P_{in}^4$$

18 $$P_{in}= c_0 + c_1r + c_2r^2 + c_3r^3 + c_4r^4$$

The values of the 15 coefficients $a_{i}$, $b_{i}$ and $c_{i}$ in Eqs. (16–18) can be found in [31]. At 1 AU the maximum thrust is of 1.56 mN, while the specific impulse is of 3582.82 s. To represent the technological limits of the thruster, $P_{in}$ has been bounded within a minimum, $P_{in,\textrm{min}}$, and a maximum, $P_{in,\textrm{max}}$, value, 80 W and 130 W respectively.

Initial and final conditions of the LOTP has been retrieved using SPICE Toolkit [1, 2] and the JPL Horizons On-Line Ephemeris System [13–15] kernels.^2^ The departure is set from the SEL2 point, while the final conditions are set to rendez-vous one of the asteroids selected as possible target of the mission. The initial value for the mass is $m_i$, the wet mass of the CubeSat, while its final value $m(t_f)$ is left free to vary to compute the fuel-optimal solution.

19 $${\textbf{r}}(t_i) = {\textbf{r}}_{L2}(t_i); \quad {\textbf{v}}(t_i) = {\textbf{v}}_{L2}(t_i) \quad \text{and} \quad m(t_i) = m_i$$

20 $${\textbf{r}}(t_f) = {\textbf{r}}_{Ast}(t_f) \quad \text{and} \quad {\textbf{v}}(t_f) = {\textbf{v}}_{Ast}(t_f)$$

As path constraints, it is imposed that the thrust can not exceed its maximum value, that the propellant mass must be positive, and that the ToF can not exceed the maximum value of 3 years, accordingly to the assumption of the mission analysis.

21 $$T-T_{max}(r) \le 0; \quad (t_f - t_i) - 1095 \, \text{days} \le 0 \quad \text{and} \quad -m(t_f) \le 0$$

The duration of the duty cycles is usually thought to preserve a standard working week. In order to keep the mission lifetime within a reasonable time span, it is preferable to not allocate less than six days for thrusting, while one day is at least required to perform all the no-thrusting operations. Thus, in this work a duty cycle of *n* = 7 days has been chosen, with *m* = 1 day for no-thrusting operations, and $(n - m)$ = 6 days employable for thrusting. Also not integer values to define *n* and *m* can be selected. For the not equally distributed time grid, with reference to Fig. 3, the choice has been $h_1 = 86{,}400\,\hbox{s} = 24 \,\hbox{h}$, $h_2 = 72{,}000\,\hbox{s} = 20 \,\hbox{h}$ and $h_3 = 14{,}400 \,\hbox{s} = 4 \,\hbox{h}.$

**Fig. 6:**
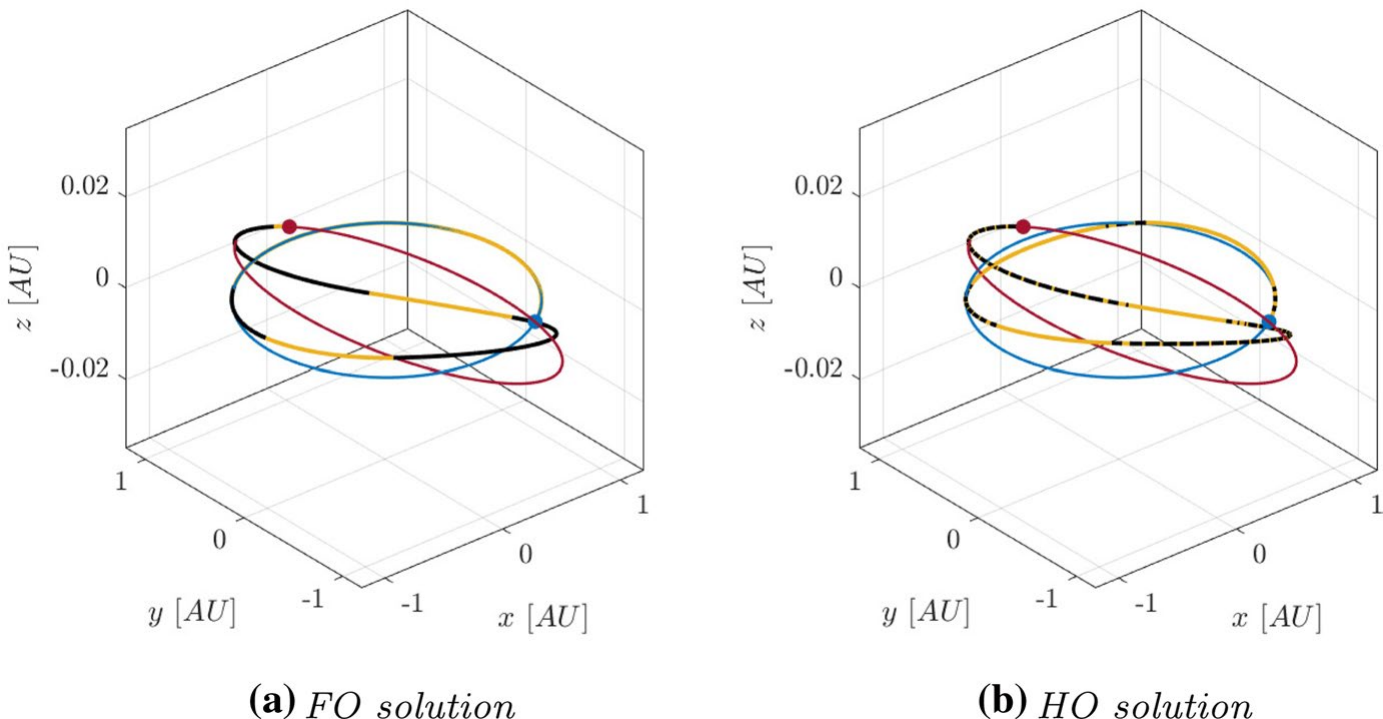
FO and HO solutions for asteroid 2014 YD in J2000 reference frame (Color figure online)

**Fig. 7:**
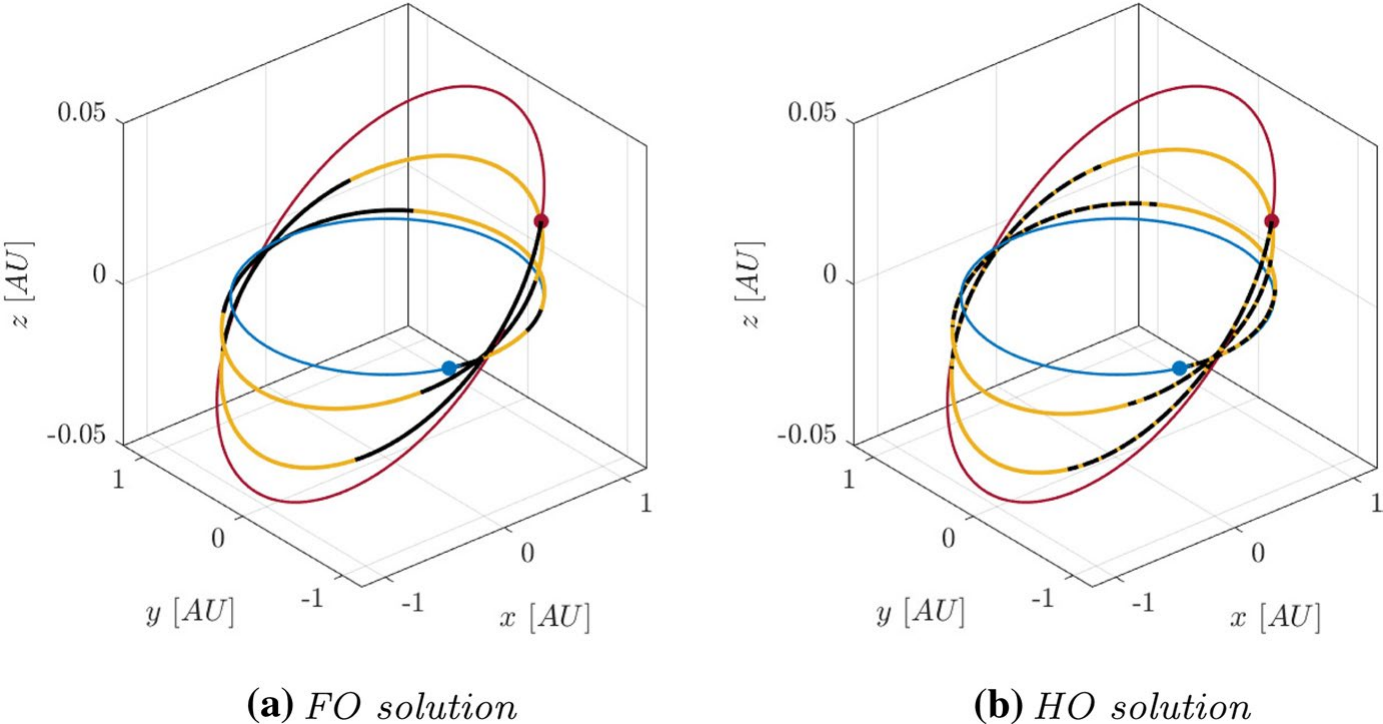
FO and HO solutions for asteroid 2011 MD in J2000 reference frame (Color figure online)

With these constraints, HDC has been used to compute several solutions towards the candidate asteroids. In this work, two of them, towards asteroids 2011 MD and 2014 YD, are presented. They are summarized in Table 2, where they are reported the Departure Date (DD), the ToF, and the value of the necessary propellant mass $m_p$ for the FO and HO solutions. The FO and HO trajectories in J2000 reference frame are reported in Figs. 6 and 7: in blue L2 Sun-Earth orbit and departure point, in red the target orbit and the rendezvous point, in yellow the coasting arcs, in black the thrusting ones.

**Table 2:** Summary of the solutions computed with HDC

Asteroid	DD	ToF (d)	Solution	${m_p}$ (kg)
2014 YD	26 Jul 2024	645	FO	1.11559
			HO	1.16563
2011 MD	05 Jun 2023	830	FO	1.26867
			HO	1.30534

In the case of the trajectory to the asteroid 2014 YD, for the fixed DD of July 26, 2024, a first search for the solution with free final time led to a solution with a ToF of 645 days. The results of the homotopies are reported in Table 3: the values of the weights for the iterations have been selected by trial and error. It has to be noted that as the weights increase going from the FO to the HO solution, the propellant mass increases too, as the solution is becoming progressively sub-optimal. However, the increase in required propellant mass $\Delta m_p$ of the HO is limited up to 4.49%.

**Table 3:** Iterations for the solution to asteroid 2014 YD

Iteration	${w_a}$	${w_b}$	${w_c}$	$\mathbf {m_p}$ (kg)	${{\Delta }} \mathbf {m_p} (\%)$
1-FO	1	1	1	1.11559	–
2	1.02	1.1	1.07	1.11666	0.10
3	1.05	1.2	1.1	1.12154	0.53
4	1.25	1.4	1.3	1.13162	1.44
5	1.35	1.5	1.4	1.13879	2.08
6	1.45	1.6	1.5	1.15296	3.35
7-HO	1.65	1.8	1.7	1.16563	4.49

**Fig. 8:**
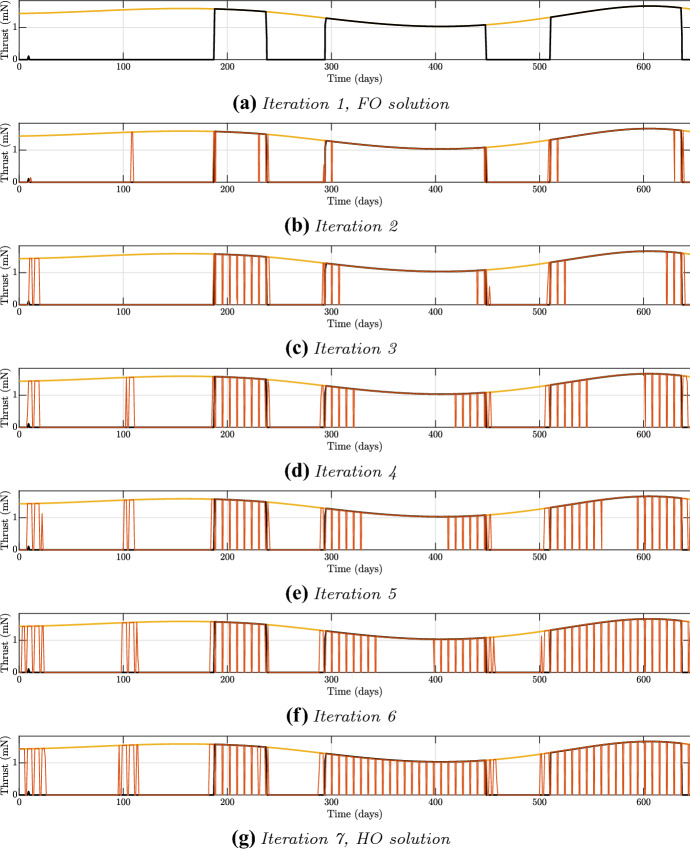
Iterations for the solution to asteroid 2014 YD

In Fig. 8, the iterations of the homotopy have been reported: the black profile is the FO solution, the red ones represent the thrusting profile at each iteration, and the yellow one represents the maximum available thrust. As expected, the additional duty cycles are located around the main thrusting blocks of the FO. It is possible to note the spreading of the thrusting blocks as the weights increase, but also the rising of two new blocks at the beginning of the transfer. The first of them is slightly observable in the FO solution, and as the homotopy proceeds it is increasingly exploited. The second one, instead, arises from scratch. It can be also noted that the thrusting block from day 300 to around 450 is the most *convenient* one, being the last one to be transformed into an OC one.

For what concerns the solution to the asteroid 2011 MD, for the fixed DD, June 5, 2023, the same considered in Sect. 5, the search for the free-time solution led to a trajectory with a value of the ToF of 830 days. The results of the homotopies are summarised in Table 4.

**Table 4:** Iterations for the solution to asteroid 2011 MD

Iteration	${w_a}$	${w_b}$	${w_c}$	${m_p}$ (kg)	${{\Delta }} {m_p}$ (%)
1-FO	1	1	1	1.26867	–
2	1.02	1.1	1.07	1.27052	0.15
3	1.05	1.2	1.1	1.27706	0.66
4	1.25	1.4	1.3	1.29306	1.92
5	1.35	1.5	1.4	1.30256	2.67
6-HO	1.45	1.6	1.5	1.30534	2.89

**Fig. 9:**
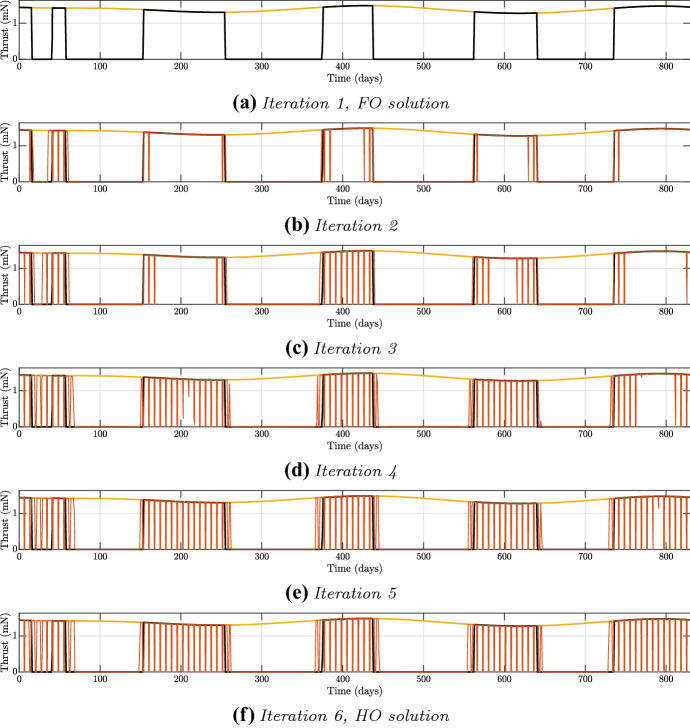
Iterations for the solution to asteroid 2011 MD

The $\Delta m_p$ of the HO is of 2.89%, and the $m_p$ required is around 1.31 kg. In Fig. 9, the iterations of the homotopy have been reported: the optimal profile is mimicked almost perfectly and no additional thrusting blocks appear. However, a sort of merging of the first two thrusting blocks into a unique operational compliant one can be observed, accordingly to the results obtained in Sect. 5.

In addition to the presented solutions, HDC has been used to compute M-ARGO trajectories towards other asteroids. The maximum $\Delta m_p$ reached is of 10.38%, with a mean value of 4.62%. It has been tested that the solutions with the highest mass increment are the ones requiring the highest number of iterations, and showing the most ‘full’ thrusting profiles, meaning that they are almost the OC time optimal—requiring the minimum ToF—solutions for their fixed DD. For these solutions, the values of the weights can increase up to 250. The computation of these solutions has been useful to have a stress-test on HDC: the algorithm works also in critical conditions where the thrusting duration is almost at its maximum allowed to be OC.

## Conclusions

In this work a new homotopic direct collocation algorithm (HDC), based on a Hermite-Simpson direct collocation and a homotopy approach, has been proposed to overcome the issue of imposing the discontinuous constraint of duty cycles to obtain OC trajectories. HDC has been tested within M-ARGO mission context, and has proved that a mapping of the fuel optimal non-OC solutions into fuel optimal and OC ones is possible. HDC trajectories show propellant mass values very close to the ones of the solutions without the OC constraint.

This algorithm opens to the possibility of modeling thrusting and coasting phases into the low-thrust control profile of each considered mission case, at the cost of a minor increase in terms of propellant mass. OC trajectories will have great impact especially on CubeSats mission analysis, having them limited propellant and power budgets. Enabling deep-space CubeSats to follow OC trajectories will also pave the way for major autonomy in space, allowing a great reduction in flight dynamics operations costs.

## Footnotes

1 https://coin-or.github.io/Ipopt/index.html (Retrieved on October 8, 2020).

2 http://ssd.jpl.nasa.gov/?horizons (Retrieved on May 11, 2020 for kernels).
